# Persistent Borrelia burgdorferi Peptidoglycan in Postinfectious Lyme Arthritis: Innate Immune Sensing and Translational Implications

**DOI:** 10.7759/cureus.118893

**Published:** 2026-10-31

**Authors:** José L. de Jesús Arguello Hernández, Emanuela K Arcos Lema, Carlos A Arragan Lezama, Rodrigo F Ale Chura, María V Fontalvo Leyton, Andrés S Estrella López

**Affiliations:** 1 Primary Health Care, Universidad Anáhuac Xalapa, Xalapa, MEX; 2 Outpatient Care, Universidad de las Américas, Quito, ECU; 3 Outpatient Care, Universidad Anáhuac Xalapa, Veracruz, MEX; 4 Primary Care, Universidad Católica de Santa María, Arequipa, PER; 5 Emergency Department, Hospital Central de la Policía Nacional, Bogotá, COL; 6 Public Health, NeoScientia Consulting Group, Quito, ECU

**Keywords:** borrelia burgdorferi infection, innate immune system, lyme arthritis, peptidoglycan, postinfectious, translational therapeutics

## Abstract

Lyme arthritis (LA) is the late articular manifestation of *Borrelia burgdorferi* infection. It is ordinarily treated successfully with appropriate antibiotic therapy. In a subset of patients, however, synovitis persists for months to years after treatment, despite presumed eradication of the spirochete. This state is termed postinfectious, or antibiotic-refractory, LA, and its cellular and molecular basis remains incompletely defined. It is distinct from post-treatment Lyme disease syndrome (PTLDS), which is defined by persistent subjective symptoms without objective joint inflammation. Attention has increasingly turned to the non-canonical peptidoglycan (PG) of *B. burgdorferi*. Three questions are central: what becomes of this material during bacterial growth, whether it persists within the joint after antimicrobial treatment, and whether it can engage innate immune sensing pathways such as NOD2. Whether such sensing occurs in the human joint has yet to be established. This narrative review synthesizes the available human, murine, and in vitro mechanistic evidence on four topics: *B. burgdorferi* PG biology, PG processing in both the bacterium and the host, the innate mechanisms of PG recognition, and the intersection of these pathways with adaptive autoimmune mechanisms in postinfectious LA. Throughout, findings established in patients are distinguished from inferences drawn from murine models and from hypotheses that remain unsupported by patient-level data. Human evidence is limited to detection of PG in synovial fluid from closely related research groups and is not independently replicated, together with genetic, immunologic, and tissue studies that do not themselves address PG. Findings such as PG tropism for the liver and NOD2 sensing derive from murine and in vitro systems. No study has shown that persistent PG or NOD2 signaling causes human postinfectious synovitis. The authors then consider the translational implications for diagnostic, prognostic, and anti-inflammatory or PG-directed strategies as hypotheses.

## Introduction and background

*Borrelia burgdorferi* *(B. burgdorferi)* is the tick-borne spirochete that causes Lyme disease in North America. Lyme arthritis (LA) is a late manifestation of infection and typically presents as monoarthritis or oligoarthritis, most frequently affecting a large joint such as the knee [1]. Although the majority of patients respond completely to oral or intravenous antibiotic therapy, a minority, estimated at about 10% in commonly cited figures [2, 3] but varying with case definition, population, and follow-up, develop postinfectious LA, defined as objective joint swelling that persists for months after recommended antibiotic therapy, with a negative synovial fluid polymerase chain reaction (PCR) for spirochetal DNA. A negative PCR argues against, but cannot by itself exclude, residual live organisms; postinfectious therefore describes a clinical state in which infection is presumed cleared, not an established mechanism. Also called antibiotic-refractory Lyme arthritis (ARLA), this condition is conceptually separate from persistent infection, which would be expected to respond to further antimicrobial therapy, and from post-treatment Lyme disease syndrome (PTLDS), which is defined by persistent subjective symptoms (fatigue, pain, cognitive complaints) without objective joint inflammation [4]. It is generally attributed to a dysregulated host immune response that persists after the pathogen [3, 4]. One question yet to be resolved in postinfectious LA is what keeps the synovium inflamed in the absence of detectable live spirochetes. One hypothesis is that non-viable bacterial remnants, such as the cell-wall peptidoglycan (PG), remain in situ in the joint and may continue to stimulate innate receptors long after the live organism has been eliminated [5]. PG is the cross-linked glycan-peptide mesh that gives bacteria their shape and integrity; its fragments are sensed by host receptors, notably nucleotide-binding oligomerization domain-containing protein 2 (NOD2), a cytosolic receptor for muramyl dipeptide that activates NF-κB signaling. The chemistry of *B. burgdorferi* PG is unusual: its stem peptide contains ornithine in place of the more common diaminopimelic acid, and rather than being recycled during growth, as in most bacteria, PG fragments are actively shed into the surrounding environment [5, 6]. PG has been detected in joint fluid but has not been shown to cause postinfectious LA; that causal link, and the involvement of NOD2 in the human joint, are hypotheses. This narrative review examines the evidence for the role of persistent *B. burgdorferi* PG in postinfectious LA, covering bacterial PG biology and shedding, human evidence for PG persistence in synovial fluid, innate sensing via NOD2 and related pattern-recognition pathways, host PG-processing enzymes and recognition proteins, and the downstream convergence of these innate pathways with adaptive autoimmune mechanisms. In all subsections, there is a clear distinction between findings supported by data from human tissues and those supported by data from only mouse models and in vitro systems, with a clear indication that causal inference in patients is hypothesis-generating and not confirmed.

## Review

Methodology

This study design is a narrative review, which did not follow a systematic review protocol, and no risk-of-bias assessment, Preferred Reporting Items for Systematic reviews and Meta-Analyses (PRISMA)-compliant flow chart, or meta-analytic synthesis was undertaken. Literature was identified only through a building-block (thematic-concept) search applied in PubMed and Scopus. Given the mechanistic, multi-domain nature of the topic, a thematic search strategy was used, and only literature that matched the scope of the review was included from 1st January 2010 to 30th September 2026 to focus on recent literature.

The literature was divided into six conceptual blocks, each searched, respectively, using Medical Subject Headings (MeSH) terms, free-text synonyms, and Boolean operators: Lyme arthritis, clinical presentation and postinfectious/antibiotic-refractory disease; biological events of *B. burgdorferi* peptidoglycan, including bacterial shedding and processing enzymes; NOD2 and related innate pattern-recognition signaling, including peptidoglycan recognition proteins; autoimmunity and molecular mimicry in Lyme arthritis; mechanistic models of *B. burgdorferi* infection and arthritis in vitro and in mice; and post-treatment Lyme disease syndrome. An example of a PubMed search string was ("Lyme arthritis" (MeSH) OR "Borrelia burgdorferi" (MeSH)) AND ("peptidoglycan" (MeSH) OR "NOD2 protein, human" (Supplementary Concept) OR "pattern recognition receptors" (MeSH) OR "autoimmunity" (MeSH)) AND ("arthritis" (MeSH) OR "synovitis" (MeSH)).

Eligibility is planned so that only articles in English and peer-reviewed databases are considered. Titles and abstracts were evaluated, and articles without information that was directly relevant to *B. burgdorferi* peptidoglycan biology, innate immune sensing, and postinfectious/antibiotic-refractory Lyme arthritis pathogenesis were not included. Articles focused on other NOD2-associated diseases (apart from arthritis, e.g., Crohn's disease genetics) or only epidemiological surveillance without mechanistic information, or articles reporting on unrelated musculoskeletal conditions were excluded. Preprints were also excluded. Thirty-nine publications were selected for inclusion based on direct relevance to the scope of the review. Evidence was synthesized narratively and grouped by evidence domain (human, murine, in vitro/structural). No quantitative synthesis was performed, and results from different studies were not pooled. The summary of evidence is presented in a tabular way to illustrate a level of evidence adapted for mechanistic literature and is descriptive, not a formal risk-of-bias assessment.

Evidence synthesis

Peptidoglycan Biology of B. burgdorferi

Peptidoglycan is the mesh-like polymer of glycan strands cross-linked by peptide bridges that provides bacteria their structural integrity, and in most Gram-negative organisms, it is sandwiched between the inner and outer membranes and continuously recycled as the cell wall is remodeled during growth. *B. burgdorferi*, an atypical diderm spirochete, departs from this pattern in ways that are directly relevant to postinfectious arthritis.

Mechanistic (Biochemical and Structural) Evidence

*B. burgdorferi* PG carries an unusual ornithine-substituted pentapeptide stem, and rather than being recycled intracellularly, PG fragments are actively shed into the extracellular environment during normal growth in culture [5, 6]. This shedding behavior, first characterized biochemically [5] and later supported using synthetic muropeptide standards that mimic the native ornithine-containing structure [6], distinguishes *B. burgdorferi* PG from that of most other pathogens and provides a plausible mechanistic basis for its persistence as a free antigen independent of viable bacteria. Several bacterial proteins govern the structural fate of this PG in biochemical and structural studies. NapA, a peptidoglycan-associated Dps homolog, binds PG in the periplasm and maintains envelope integrity; loss of NapA destabilizes the cell wall, and NapA-linked PG released in outer membrane vesicles augments interleukin-17 (IL-17) production beyond that induced by PG alone in cell-culture assays [7]. The lytic transglycosylase encoded by BB0259 creates the pores required for periplasmic flagellar assembly, and its enzymatic activity, defined by cryo-electron tomography, is essential for normal flagella architecture, illustrating that PG remodeling is functionally coupled to motility as well as cell-wall maintenance [8]. Structural work on the outer membrane protein OmpA has further shown, by X-ray crystallography, that its C-terminal domain adopts a PG-binding conformation homologous to that of other Gram-negative pathogens, anchoring the outer membrane to the cell wall [9].

Murine (Animal Model) Evidence

Two of these PG-processing systems have additionally been validated in live-animal infection models. A separate accessory protein, Borrelia peptidoglycan-interacting protein (BpiP), binds PG directly and contributes to bacterial fitness against host-derived antimicrobial factors; in C3H/HeN mice, mutants lacking BpiP show reduced tissue burden and increased susceptibility to vancomycin, complement, and antimicrobial peptides [10]. Similarly, deletion of a putative PG carboxypeptidase (DacA homolog) that governs stem-peptide cross-linking alters PG architecture and tissue tropism in mice without materially changing in vitro growth and produces near-complete attenuation of murine Lyme arthritis, directly linking PG structural chemistry to arthritogenic potential in this animal model [11]. Collectively, these predominantly in vitro and murine studies establish that *B. burgdorferi* possesses a dedicated, chemically distinctive PG-processing machinery whose products are shed rather than internally recycled; none of this structural and enzymatic characterization has yet been performed on bacteria recovered directly from human joints.

Evidence for persistent peptidoglycan in postinfectious Lyme arthritis

The clinical relevance of this bacterial biology rests on direct human evidence that *B. burgdorferi* PG is detectable in the joints of patients with postinfectious LA after antibiotic treatment. The human and murine animal model evidence has been analyzed separately below.

Human Evidence

Jutras et al. identified *B. burgdorferi *PG in synovial fluids of 32 out of 34 patients with LA using a novel technique; some patients had previously been treated with oral and intravenous antibiotics; the patients displayed an immunoglobulin G response to PG, which was greater in the synovial fluid than in paired serum, and synovial fluid containing *B. burgdorferi* PG had also contained pro-inflammatory cytokines that were detected in in vitro human PBMC stimulated with purified PG [5]. This study is the principal human evidence that PG is found in the inflamed joint in LA, and it has important limitations. The cohort was small and drawn from a limited number of centers, and it included patients sampled at different stages of disease and treatment, so it does not show PG persistence specifically in antibiotic-refractory disease. It has not been independently replicated, and the sensitivity and specificity of the detection method and its validation across different patient groups have not been addressed. Detection shows that the antigen is present, not that it is pathogenic, and no study has compared PG burden between patients whose arthritis resolved and those whose arthritis persisted. It should be read as an association that motivates, rather than demonstrates, the persistent-antigen hypothesis.

Murine/Animal Model Evidence

Systemic administration of *B. burgdorferi *PG induced acute arthritis in BALB/c mice in the absence of live spirochetes, identifying shed PG as a likely contributor to joint inflammation in this model [5]. Subsequent murine studies have since confirmed this, in that they have looked at the location and fate of the shed PG. In real-time in vivo tracking experiments, McClune and co-workers have shown that *B. burgdorferi* PG, due to its unique chemical properties, specifically targets liver tissue and was found to accumulate there in mice, where *B. burgdorferi *PG is engulfed and retained by the Kupffer cells and hepatocytes for several weeks, which results in alteration in the systemic protein and transcriptomic profile, similar to how it is observed in patients with chronic postinfectious illness, but this liver-specific accumulation has not yet been directly confirmed in human liver tissue, and any relationship to post-treatment Lyme disease syndrome is speculative [12]. Consistent with this, a DacA-deficient *B. burgdorferi* strain with altered PG stem-peptide chemistry and cross-linking showed shifted tissue tropism, altered interaction of the PG sacculus with the periplasmic protein p83/100 previously implicated in arthritogenicity, and near-complete loss of joint tropism, indicating that PG architecture itself shapes arthritogenic potential in this model [11]. Among these data, the findings of Jutras et al. [5] in human synovial fluid provide the principal human evidence that PG can be detected in the joint in LA, a finding not yet independently replicated, while the murine tracking and structural studies offer insight into how PG chemistry may influence tissue distribution and retention; the latter findings have not been confirmed in humans.

Innate immune sensing: NOD2 and peptidoglycan recognition proteins

Bacterial PG fragments are inert until they can be sensed by a host. Moreover, the key cytosolic sensor, the nucleotide-binding oligomerization domain-containing protein 2 (NOD2), recognizes the minimal degradation fragment, which is the muramyl dipeptide (MDP) found in both Gram-positive and Gram-negative bacteria [13].

NOD2 Signaling: Mechanistic Pathway and Human Disease-Analogue Evidence

More than 30 host proteins have been shown to interact with NOD2 and modulate its signaling. Upon MDP binding, NOD2 recruits receptor-interacting serine/threonine kinase 2 (RIP2/RIPK2), triggering activation of nuclear factor-kappa B (NF-κB) and mitogen-activated protein (MAP) kinase pathways, processing of pro-interleukin-1β, induction of autophagy, and production of antimicrobial peptides [13]. The best-known clinical associations of NOD2 are with disease analogs rather than with Lyme arthritis itself: variants in the leucine-rich repeat region are linked to Crohn disease, while a gain-of-function variant in the nucleotide-binding domain causes Blau syndrome, a monogenic autoinflammatory disorder with granulomatous arthritis [14]. These disorders show that abnormal NOD2 signaling can produce chronic joint inflammation in humans. They do not establish that PG sensing by NOD2 drives arthritis, but they make it biologically plausible that persistent *B. burgdorferi* PG in the joint could engage the same pathway, a hypothesis that remains untested in patients. NOD2 activation by B. burgdorferi PG has not been examined in human synovial tissue.

Peptidoglycan Recognition Proteins: Murine and Structural Evidence

The mammalian peptidoglycan recognition protein (PGLYRP) family provides a second line of PG surveillance, mostly extracellular. The number of PGLYRPs is four in humans: two are present at body surfaces and in secretions, PGLYRP1 is present in polymorphonuclear leukocytes, and PGLYRP2 is an enzymatic N-acetylmuramoyl-L-alanine amidase that is able to hydrolyze PG; several PGLYRPs are directly bactericidal in biochemical assays, and PGLYRPs can regulate inflammation in arthritic joints in ways beyond their antimicrobial functions [15]. Using a systems-level, yeast-display screen of human secreted and extracellular proteins against multiple *B. burgdorferi* isolates, Gupta et al. identified peptidoglycan recognition protein 1 (PGLYRP1), a human innate immune protein, as binding directly to purified *B. burgdorferi *peptidoglycan and exhibiting borreliacidal activity in vitro; PGLYRP1-deficient mice subsequently showed increased spirochete burden in the heart and joints, splenomegaly, and signs of immune dysregulation [16]. This murine and in vitro study, therefore, gives rise to a number of very important, contemporary, and unanswered translation questions that focus on PGLYRP1 or other PGLYRPs as a player in the persistence or inflammatory potency of the PG specifically in the joints of humans with LA. However, human relevance has yet to be established.

Host Peptidoglycan-Processing Mechanisms and Bacterial-Host Interface

The persistence of arthritogenic PG in the joint likely reflects a balance between bacterial shedding, structural resistance to degradation, and the efficiency of host clearance mechanisms. However, the outer membrane vesicles (OMVs) appear to be one vehicle for extracellular PG dissemination.

Mechanistic and In Vitro Evidence

Purified *B. burgdorferi* OMVs contain known antigenic markers, including outer surface proteins OspA and OspC, the joint-associated marker p39, and peptidoglycan itself; although co-culture of these OMVs with cultured human non-immune cell lines did not induce cytotoxicity in vitro, the authors proposed that OMVs may function as decoys for the host immune system and as a vehicle allowing persistent antigens, including PG, to remain in host tissue over prolonged periods, a hypothesis not yet directly tested in synovial tissue [17]. Separately, the chemical synthesis of defined ornithine-containing muropeptide fragments that mimic native *B. burgdorferi* PG chemistry has enabled more rigorous dissection, in cell-based assays, of which structural motifs drive innate immune activation, providing reagents for future mechanistic studies rather than direct new pathogenic findings on their own [6]. On the therapeutic side, comparative in vitro and murine antibiotic-killing studies show that vancomycin, which acts on cell-wall PG synthesis, is more effective than ceftriaxone at killing *B. burgdorferi *in vitro, including in the stationary growth phase, an unexpected finding attributed to ongoing PG synthesis in non-dividing spirochetes; in a severe combined immunodeficient (SCID) mouse model, doxycycline failed to clear infection, whereas both ceftriaxone and vancomycin cleared it equally effectively [18]. These studies address bacterial killing in culture and in immunodeficient mice, not postinfectious inflammation.

Integrated immunopathogenesis: from innate sensing to autoimmune synovitis

Postinfectious LA has best been understood as a result of a normal, subsequently dysregulated innate immune response. However, it doesn't resolve after spirochetal killing but rather becomes chronic, partly autoimmune synovitis.

Human Genetic Susceptibility Evidence

This structure is supported by the genetic studies in the patient cohorts; a variant in Toll-like receptor 1 (TLR1-1805GG), a molecule that normally functions as an innate immune sensor of *B. burgdorferi* lipoproteins, is more common in patients that develop postinfectious LA than in those in whom the arthritis resolves with antibiotic treatment [19], and functional studies in patient-derived peripheral blood mononuclear cells (PBMCs) demonstrate that PBMCs with the variant show decreased innate immune tolerance typically induced by repeated stimulation by* B. burgdorferi*, including greater hyperinflammatory responses [19]. Similarly, polymorphisms affecting the late cornified envelope 3 (LCE3) gene are enriched in host genetic susceptibility and correlate with increased levels of systemic T-helper 17 (Th17) cytokines and decreased levels of regulatory interleukin-27 (regulatory Il-27) in postinfectious compared with antibiotic-responsive LA [20].

Human Synovial Tissue and Fluid Evidence

Interferon-gamma (IFN-γ) is abundant in the synovial tissue and fluid of patients with LA. When fibroblast-like synoviocytes (FLS) obtained from these patients are stimulated in vitro with IFN-γ, alone or together with *B. burgdorferi*, they adopt a highly inflammatory phenotype expressing MHC class II and co-stimulatory molecules and present peptides, including one derived from the Lyme autoantigen fibronectin-1 [21, 22]. In the matched serum, downstream Th17 responses correlated with control of *B. burgdorferi* IgG responses early in infection but demonstrated a correlation with autoantibodies to endothelial cell growth factor, matrix metalloproteinase 10, and apolipoprotein B-100 late in disease, presenting an autoimmune phenotype [23]. Kanjana et al. performed unbiased immunopeptidomics on human synovial tissue to find that, in 58% of postinfectious LA patients studied, autoreactive responses to CD4+ T-cell epitopes derived from extracellular matrix proteins, including those of fibronectin-1, laminin B2, and collagen Vα1, were enriched compared with controls; of note, the authors proposed an epitope-spreading model rather than classic molecular mimicry to explain the infection-induced autoimmunity, a distinction that undermines earlier simpler mimicry-based hypotheses [24]. Complementary mass-spectrometry-based studies have been conducted to identify other self-peptides that are directly presented by HLA-DR on human synovial tissue, synovial fluid, and peripheral blood samples [25]. This autoimmune amplification is a quantifiable downstream effect in human synovial disease: The level of IgG4 autoantibodies against Lyme disease-related autoantigens is directly proportionate to the amount of obliterated microvascular changes and fibrotic changes of refractory synovial tissue, and the glycosylation signature of antibodies in AB-resistant patients is more pro-inflammatory than in AB-responsive patients [26,27]. CD8+ and γδ (double-negative) T cells are enriched in the synovial fluid of patients with postinfectious LA compared to those with antibiotic-responsive LA and frequently express granzyme and perforin; in synovial tissue sections, CD8+ T cells (but only rarely γδ T cells or NK cells) were seen intermixed with CD4+ T cells around blood vessels, providing a potential cellular mechanism, mediated primarily by CD8+ T cells, for the microvascular damage observed in some patients [28]. MicroRNA profiling of the patient-derived synovial fluid and tissue also reveals a shift of miRNAs from an infection-associated, neutrophil-enriched miRNA signature during active disease towards an inflammatory, proliferative miRNA signature (miR-146a, miR-155, miR-142, miR-17-92) following antibiotic therapy, in a similar fashion to that observed in rheumatoid arthritis (RA) [29].

Murine Mechanistic Evidence

In parallel murine experiments, FLS co-cultured with OT-II T cells acted as inducible antigen-presenting cells driving antigen- and CD40-dependent CD4+ T-cell proliferation, and stimulation with* B. burgdorferi *PG enhanced this FLS-mediated T-cell activation; the corresponding experiment has not been performed with human T cells [22]. Murine immunopeptidomic analysis has also revealed that MHC class II epitopes of the Lyme autoantigen apo-lipoprotein B-100 and one of the *B. burgdorferi* chemotaxis proteins, Mcp4, are detected during infection in mice; the limited number of identified immunogenic bacterial epitopes has been suggested to support the concept that CD4+ T immune responses to* B. burgdorferi* as a whole may be actively suppressed during infection and that the postinfectious autoinflammatory response may be more directed at host-derived antigens as opposed to bacterial ones, although the suppression has been observed in mice but not directly confirmed in human T cells [30]. The comprehensive reviews of this dual human and murine literature are unified by an emerging model wherein a hyperactive innate and Th1/Th17 response induced and propagated by persistent PG and other bacterial release is followed by "epitope spreading" and relative dysfunction of regulatory mechanisms (interleukin-10, Treg), ultimately producing a self-perpetuating autoinflammatory synovitis that mechanistically resembles other chronic inflammatory arthritides such as rheumatoid arthritis [31, 32]. It should be emphasized, however, that the majority of the cellular and molecular mechanisms involved in this integrated model, such as the role of FLS antigen presentation and the findings of cytotoxicity, are from human synovial tissue and fluid studies conducted in a limited number of research centers, and that no formal direct demonstration has yet shown that sensing of PGs by NOD2 in humans is the immediate trigger activating this signaling cascade, rather than one of a variety of inputs to an innate pathway.

Clinical and translational implications

If persistent PG contributes to postinfectious LA, there are several implications for patient management and for the development of new therapies. The synthesis below explains the human clinical and epidemiological evidence and emerging ex vivo and translational models. 

Human Clinical and Epidemiologic Evidence

In a retrospective national cohort of PCR-confirmed LA, 34% of treated patients (12/35) developed persistent synovitis lasting at least two months (median three months, range 2-16) despite appropriate antibiotic therapy; in the subset who went on to develop systemic oligo- or polyarthritis in previously unaffected joints, repeat synovial fluid PCR was negative, indicating inflammation without evidence of persistent infection [33]. In a multicenter pediatric case-control study, factors associated with an antibiotic-refractory course included age of 10 years or older, prolonged arthritis at diagnosis, knee-only involvement, and paradoxical worsening after starting antibiotics [34]. Taking into account human cohort studies, a systematic review of potential LA biomarkers identified Th17-pathway cytokines and a panel of microRNAs as the most promising candidates for predicting LA, but evidence for each of them remains scarce, and no individual biomarker is currently ready for clinical use [35]. The deposition patterns of antibody and complement in patient joint fluid also differentiate antibiotic-refractory from antibiotic-responsive LA and could lead to earlier immunologic stratification at diagnosis [36]. The clinical importance of this course of action is underlined by a population-level administrative claims study showing a higher incidence of new autoimmune inflammatory arthritis (including rheumatoid arthritis, psoriatic arthritis, and spondyloarthritis) in the two-year period after Lyme disease than after influenza [37], corroborating an earlier single-center case series that first described this pattern of new-onset autoimmune inflammatory arthritis following Lyme disease [38]. Distinguishing persistent infection from postinfectious inflammation is clinically important because prolonged antibiotic courses carry toxicity without added benefit once the spirochete has been cleared; in the pediatric cohort, children with antibiotic-refractory disease received more antibiotics and experienced more treatment-associated adverse events (37% vs 15%) and resultant hospitalization (6% vs 1%) than those who responded [34].

Emerging Human Ex Vivo and Translational Models

Ex vivo human synovial tissue models offer a route to study these processes directly in patient tissue. In one such platform, synovial biopsies obtained from patients undergoing total joint replacement were exposed to *B. burgdorferi* and showed dose- and time-dependent loss of synovial intimal lining integrity, altered subcellular localization of the tight-junction protein ZO-1, reduced osteoprotegerin secretion, and increased interleukin-10, with sustained expression of the M2 macrophage marker CD206 in joint-lining macrophages despite infection; the authors emphasized that a causative link between these changes and disease has yet to be established [39]. This system models acute spirochetal exposure rather than postinfectious disease, but it illustrates a platform in which PG-targeted or macrophage-directed interventions could in principle be tested in human tissue rather than by extrapolation from murine models.

Looking forward, the detection of PG as a persistent antigen in human joint fluid raises the possibility of developing PG-based diagnostic assays to distinguish postinfectious LA from ongoing infection or from unrelated autoimmune arthritis. Preclinical studies have compared antibiotics against B. burgdorferi in culture and in SCID mice [18]. Vancomycin killed the organism more effectively than ceftriaxone in vitro, including in stationary phase, and in SCID mice doxycycline failed to clear infection while ceftriaxone and vancomycin did so. These data concern bacterial killing in culture and in mice without adaptive immunity. SCID mice cannot model postinfectious autoimmune synovitis, and no study has tested whether antibiotic choice or intensity alters the risk of postinfectious LA. These findings offer no basis for changing current treatment, and prolonged antibiotic exposure carries toxicity without demonstrated benefit once the spirochete is cleared [34].

Gaps in knowledge and future research directions

While there have been significant advances, there are some key gaps. The first is that, to date, there is no evidence that *B. burgdorferi *PG signals directly via NOD2 in human synovial tissue, and the association between NOD2 and postinfectious LA is inferential from diseases in which NOD2 is involved, but not from mechanism studies done in patients. Second, while the mouse studies show that PG tends to localize in the liver and cause signatures in the bloodstream that mimic postinfectious illness, this has not been confirmed in human tissues and should be thought of as a hypothesis if applied to post-treatment Lyme disease syndrome [4, 12]. Third, the effects of PGLYRP1 on *B. burgdorferi *burden and inflammatory dysregulation in mice have been reported, but no studies have examined the effect of the other PGLYRP family members on PG persistence or clearance in the human joint. Fourthly, the relative role of epitope spreading versus classical molecular mimicry in relation to the autoimmune responses in postinfectious LA is incompletely answered, and the field has not yet shifted to an entirely different model. Last but not least, there is no biomarker panel or PG-based diagnostic assay that has been specifically validated prospectively for differentiation of postinfectious LA from persistent infection, post-Lyme autoimmune arthritis, and unassociated inflammatory joint disease. Future studies should focus on direct assessment of NOD2/RIP2 pathway activation in human LA synovial tissue and on prospective studies to further validate PG as a biomarker using the Th17-cytokine and microRNA candidates recently identified.

Limitations

This review has several limitations that should be considered when interpreting its findings. It is a narrative review without a formal risk-of-bias assessment, so study selection and weighting involve author judgment. Only two databases were searched, only English-language peer-reviewed articles were included, and preprints were excluded, which is usually expected in a narrative review. A single research network contributes much of the PG-specific evidence, and the human evidence for PG persistence rests principally on a single study, which limits independent corroboration. The included studies differ widely in design, species, and model, and could not be compared quantitatively. Table 1 separates findings shown in humans from those shown only in animal or in vitro systems and from untested hypotheses. The conclusions about PG persistence and innate sensing should therefore be read as a synthesis of current hypotheses and not as a confirmatory mechanism.

**Table 1:** Summary of findings status in preclinical and human evidence BpiP - Borrelia peptidoglycan-interacting protein; CD4+/CD8+ - cluster of differentiation 4/8 (T-cell co-receptors); DacA - D-alanyl-D-alanine carboxypeptidase A; FLS - fibroblast-like synoviocytes; IgG/IgG4 - immunoglobulin G/immunoglobulin G subclass 4; LA - Lyme arthritis; LCE3 - late cornified envelope protein 3; microRNA/miRNA - micro-ribonucleic acid; NapA - peptidoglycan-associated protein NapA; NOD2 - nucleotide-binding oligomerization domain-containing protein 2; PG - peptidoglycan; PGLYRP / PGLYRP1 - peptidoglycan recognition protein / peptidoglycan recognition protein 1; PTLDS - post-treatment Lyme disease syndrome; RIP2 - receptor-interacting serine/threonine-protein kinase 2; SCID - severe combined immunodeficiency; TLR1 - toll-like receptor 1; γδ - Gamma delta (T-cell receptor subunit)

Evidence status	Findings	Key limitation
Shown in humans	PG can be detectable in LA synovial fluid [5]; IgG to PG higher in synovial fluid than serum [5]; postinfectious LA frequency and pediatric risk factors [33,34]; excess autoimmune arthritis after Lyme disease [37,38]; TLR1 and LCE3 genetic associations [19,20]; autoreactive CD4+ T cells to extracellular-matrix proteins [24]; IgG4 autoantibodies, CD8+/γδ cytotoxic T cells, microRNA signatures [26–29]	PG data from closely related groups; small cohorts; not independently replicated. Other human findings describe the autoimmune phenotype and do not test PG.
Shown only in animals or in vitro	PG induces arthritis in mice [5]; PG tropism for liver [12]; PG architecture (DacA) shapes joint tropism [11]; BpiP and NapA effects [7, 10]; PGLYRP1 deficiency raises spirochete burden [16]; PG-enhanced FLS–T cell activation in murine co-culture [22]; antibiotic comparisons in SCID mice [18].	Murine PG chemistry, tissue tropism, and immunity differ from human ones; SCID mice lack adaptive immunity.
Hypothesis, untested	PG causes persistent human synovitis; NOD2/RIP2 activation by PG in human synovium; PG liver reservoir relevant to PTLDS; PGLYRP family role in human joint PG clearance; PG-based diagnostic assay; any antibiotic strategy to reduce PG burden	No human or prospective data.

Summary of the level of evidence

Thirty-nine studies were included in this narrative synthesis. Table 2 summarizes each study by design (human, murine, or mechanistic/in vitro) and assigns a level of evidence adapted for mechanistic and translational literature so that the clinical applicability of each finding can be evaluated.

**Table 2:** Summary of key evidence on B. burgdorferi peptidoglycan and innate immune sensing in postinfectious Lyme arthritis Level of evidence adapted for mechanistic/translational literature (not therapeutic-trial evidence): I - systematic review/meta-analysis; II - human cohort, case-control, or genetic-association study; III - human ex vivo/mechanistic study or narrative review; IV - murine/animal infection model; V - in vitro, biochemical, or structural study. This scheme is descriptive and is not a validated hierarchy or a risk-of-bias assessment. ApoB-100 - apolipoprotein B-100; ARLA - antibiotic-refractory Lyme arthritis; BB0259 - Borrelia burgdorferi gene BB0259, encoding a peptidoglycan lytic transglycosylase; BpiP - Borrelia peptidoglycan-interacting protein; DacA - D-alanyl-D-alanine carboxypeptidase A homolog; ECM - extracellular matrix; HLA-DR - human leukocyte antigen–DR isotype; IFN-γ - interferon-gamma; IgG - immunoglobulin G; IgG4 - immunoglobulin G subclass 4; LA - Lyme arthritis; LCE3 - late cornified envelope 3; Mcp4 - methyl-accepting chemotaxis protein 4; MHC - major histocompatibility complex; NapA - peptidoglycan-associated protein NapA; NOD2 - nucleotide-binding oligomerization domain-containing protein 2; OmpA - outer membrane protein A; PCR - polymerase chain reaction; PG - peptidoglycan; PGLYRP - peptidoglycan recognition protein; Th17 - T-helper 17; TLR - Toll-like receptor.

Review section / topic	Study type / references	Level of evidence
Lyme borreliosis overview / staging	Narrative review [1, 2]	III
Postinfectious LA / ARLA overview	Narrative review perspective/review [3, 4]	III
PCR-positive LA outcomes cohort	Retrospective cohort [33]	II
Pediatric antibiotic-refractory LA	Case-control study [34]	II
Incidence of autoimmune arthritis post-Lyme	Retrospective claims cohort [37]	II
Systemic autoimmune arthritides post-Lyme	Case series [38]	III
PG chemical structure / shedding, muropeptide synthesis	Human cohort + murine model biochemical synthesis [5]	II
NapA, a peptidoglycan-associated protein	In vitro / structural (murine mutant) [7]	V
BB0259 lytic transglycosylase	In vitro / structural [8]	V
OmpA PG-binding domain structure	Structural (crystallography) [9]	V
BpiP is a PG-interacting protein.	Murine infection model [10]	IV
DacA carboxypeptidase / PG architecture	Murine infection model [11]	IV
PG persistence in human synovial fluid	Human cohort study [5]	II
PG tissue tropism and systemic persistence	Murine model [12]	IV
NOD2 signaling pathway mechanism	Mechanistic review [13]	III
NOD2 structure, function, and disease associations	Narrative review [14]	III
Peptidoglycan recognition proteins (PGLYRP1-4)	Narrative review [15]	III
PGLYRP1 host interaction / borreliacidal activity	Murine model + in vitro screen [16]	IV
Outer membrane vesicle antigen content	In vitro study [17]	V
Synthetic muropeptide fragment library	Biochemical synthesis [6]	V
Vancomycin efficacy against B. burgdorferi	In vitro + murine model [18]	IV
TLR1 polymorphism and immune tolerance	Human genetic/functional study [19]	II
LCE3 locus variants and Th17 responses	Human genetic association study [20]	II
IFN-γ-driven synoviocyte differentiation	Human ex vivo / in vitro study [21]	III
HLA-DR+ synoviocytes as antigen-presenting cells	Human ex vivo + murine co-culture [22]	III
Th17 cytokine responses in LA	Human cohort study [23]	II
Autoimmunity to synovial ECM proteins	Human immunopeptidomics cohort [24]	II
HLA-DR-presented self-peptides	Human clinical sample analysis [25]	II
IgG4 autoantibodies and synovial pathology	Human cohort study [26]	II
IgG antibody glycosylation profiles	Human cohort study [27]	II
Cell-mediated cytotoxicity in synovium	Human cohort study [28]	II
MicroRNA expression in synovial fluid/tissue	Human cohort study [29]	II
MHC class II epitope mapping (ApoB-100, Mcp4)	Murine immunopeptidomics [30]	IV
Infection, inflammation, and autoimmunity in LA	Narrative review [31]	III
Innate-adaptive immune interplay in LA	Narrative review [32]	III
Biomarkers of antibiotic-refractory LA	Systematic review [35]	I
Antibody/complement profiles distinguishing ARLA	Human cohort study [36]	II
Ex vivo synovial macrophage response model	Human ex vivo tissue model [39]	III

## Conclusions

The peptidoglycan of *B. burgdorferi* is chemically unusual and is shed rather than recycled as the bacterium grows in culture. Although human data are limited, it has been detected in the joint fluid of most Lyme arthritis patients after antibiotic treatment, and systemic administration alone can induce arthritis in mice. The antigen may be a likely target for innate pattern-recognition mechanisms, such as the NOD2 receptor and the peptidoglycan recognition protein family, which are independently involved in the pathogenesis of chronic inflammatory arthritis in other human diseases. After initial innate activation, downstream adaptive pathways (Th17 and interferon-gamma-dominated responses) may underlie epitope spreading to synovial extracellular matrix autoantigens, coupled with cytotoxic and humoral effector mechanisms, which may maintain synovitis despite long-term eradication of the pathogen. PG has been detected in the joints of patients, some of whom had previously been treated; no study has compared PG levels between patients whose arthritis resolves and those in whom it persists, and none has shown that PG drives the persistent disease; the further suggestion that NOD2 signaling is the intermediate mechanistic link between persistent PG and human synovitis is an extrapolation based on shared features from murine and disease-analog conditions and should be treated as such in the interpretation and design of future mechanistic and translational studies in this area.
